# Secondary syphilis: a nodular presentation with granulomatous inflammation—a case report

**DOI:** 10.1093/omcr/omaf277

**Published:** 2025-12-26

**Authors:** Maryam Ghaleb, Ouiame El jouari, Kaoutar Benchekroun, Salim Gallouj

**Affiliations:** Dermatology Department, Mohammed VI University Hospital, Faculty of medicine and pharmacy, Abdelmalek Essaâdi University, Route de Rabat Km 17 BP 398, Gzinaya, Tangier, Morocco; Dermatology Department, Mohammed VI University Hospital, Faculty of medicine and pharmacy, Abdelmalek Essaâdi University, Route de Rabat Km 17 BP 398, Gzinaya, Tangier, Morocco; Dermatology Department, Mohammed VI University Hospital, Faculty of medicine and pharmacy, Abdelmalek Essaâdi University, Route de Rabat Km 17 BP 398, Gzinaya, Tangier, Morocco; Dermatology Department, Mohammed VI University Hospital, Faculty of medicine and pharmacy, Abdelmalek Essaâdi University, Route de Rabat Km 17 BP 398, Gzinaya, Tangier, Morocco

**Keywords:** granulomatous inflammation, nodular syphilis, secondary syphilis, *Treponema pallidum*, case report

## Abstract

Syphilis, caused by *Treponema pallidum*, is a re-emerging infectious disease with diverse clinical manifestations. While primary syphilis often goes unnoticed, secondary syphilis is characterized by systemic symptoms and variable skin lesions, earning it the epithet ‘the great imitator’. Histologically, secondary syphilis typically shows a plasma cell-rich dermal infiltrate, whereas granulomatous inflammation is rare. This report describes a case of nodular secondary syphilis with granulomatous inflammation in a 22-year-old woman who presented with persistent erythematous nodules on the face. Initial mismanagement with corticosteroids delayed the diagnosis, which was later confirmed by histopathology and positive serologic tests. Treatment with benzathine penicillin G led to complete resolution of the lesions. The case highlights the importance of recognizing atypical presentations of syphilis, especially granulomatous forms, in the differential diagnosis. Serologic testing remains essential for confirmation, and benzathine penicillin is the treatment of choice for all stages of syphilis.

## Introduction & Objectives

Syphilis is an infectious disease caused by the bacterium *T. pallidum*, a type of microaerophilic spirochete transmitted through close contact with infected individuals. Although the incidence of syphilis declined markedly in the late 20th century, recent years have witnessed a resurgence worldwide [1].

The early stage of syphilis, characterized by a painless ulcer known as a chancre, frequently goes unnoticed, and many patients present only during the secondary stage, when a widespread rash develops. Approximately 25% of untreated individuals progress to the secondary stage. In addition to the rash, nonspecific systemic symptoms such as fever, headache, and fatigue are common [2].

Because of its wide range of skin manifestations, secondary syphilis has been termed ‘the great imitator’. Its histopathologic features are similarly diverse, posing diagnostic challenges for both dermatologists and pathologists. The classic histologic pattern includes a plasma cell-rich infiltrate in the dermis along with endothelial swelling or proliferation, but no single finding is pathognomonic. Granulomatous inflammation is particularly rare [3, 4].

This report presents a case of nodular secondary syphilis with granulomatous inflammation and briefly reviews the literature on this uncommon histopathologic variant.

## Case report

A 22-year-old woman presented with erythematous, mildly painful, progressively enlarging nodules on her forehead, nose, and chin for the past 18 months, accompanied by headaches and fatigue. She showed minimal improvement following systemic corticosteroid therapy.

**Figure 1:**
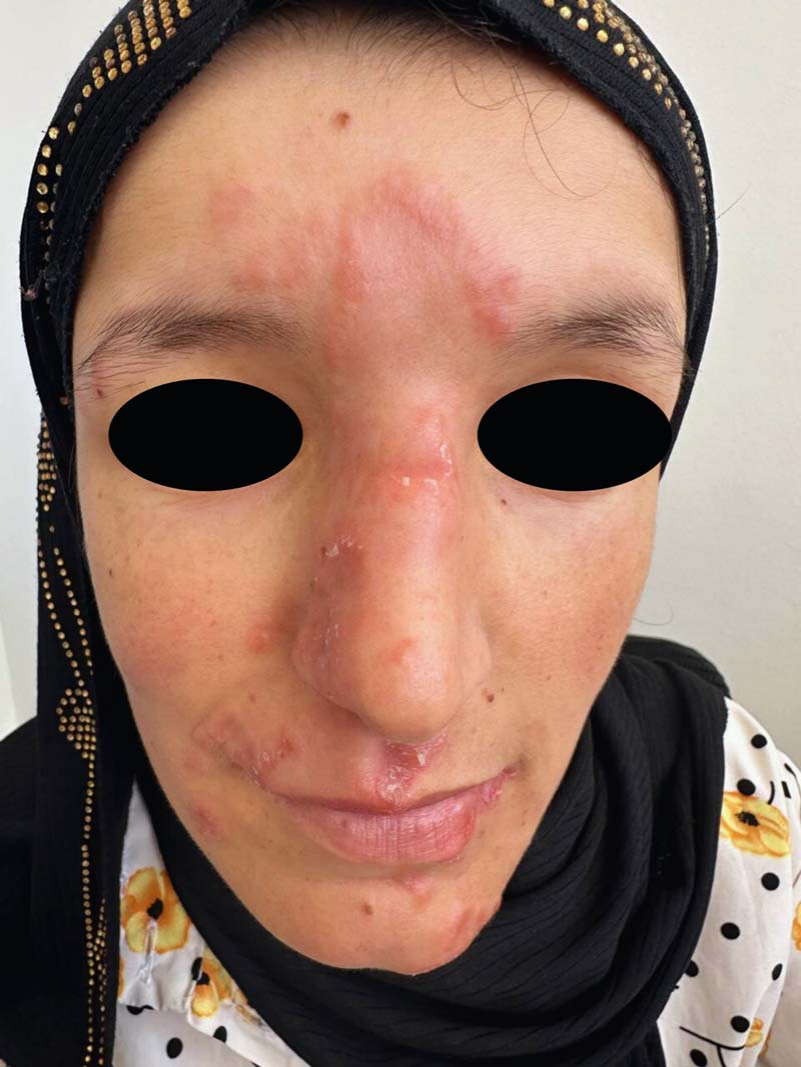
Multiple well-defined papules and nodules, 3–15 mm in size, brown-red in color with focal orange areas on the forehead, and several others on the nose and chin.

Physical examination revealed multiple, well-defined brown-red papules and nodules measuring 3 to 15 mm in diameter, with focal orange areas on the forehead, and several others on the nose and chin (Fig. 1).

**Figure 2:**
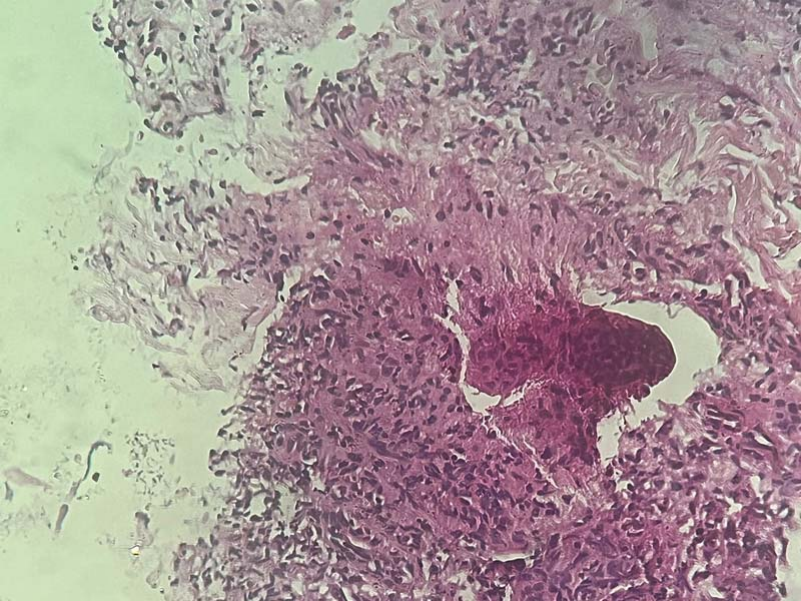
Histopathology of the mandibular lesion showing a dense dermal granulomatous infiltrate composed of lymphocytes and numerous plasma cells.

A punch biopsy of one lesion revealed an atrophic epidermis with orthokeratotic hyperkeratosis. The granular layer was reduced, and the epidermal papillae were slightly diminished. The basal layer displayed interface changes with apoptotic keratinocytes. The superficial dermis was fibro-collagenous, with foci of fibro-edematous regression, containing a diffuse inflammatory infiltrate organized in a deep granulomatous pattern composed of lymphoplasmacytes and epithelioid cells (Fig. 2). No giant cells or caseous necrosis were seen, and neither asteroid nor Schaumann bodies were present.

Periodic acid–Schiff staining revealed basal layer thickening, with no mucin deposits on Alcian blue stain and no acid-fast bacilli on Ziehl–Neelsen stain. Immunohistochemistry was not performed due to limited technical resources.

Serologic testing showed a reactive venereal disease research laboratory (VDRL) test with a titer of 1/32 and a positive *T. pallidum* hemagglutination assay (TPHA) with a titer of 1/2560. These findings were consistent with secondary syphilis.

**Figure 3:**
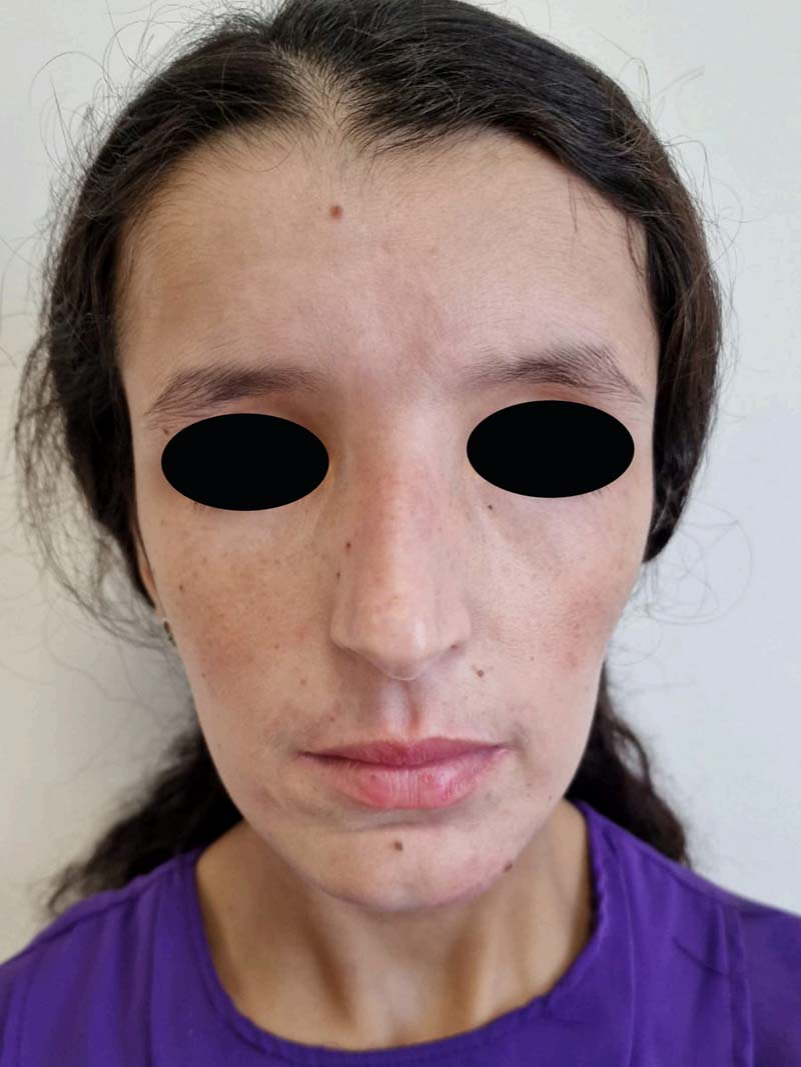
Complete resolution after three doses of intramuscular benzathine penicillin (2.4 million units each).

The patient was treated with three weekly intramuscular injections of benzathine penicillin G (2.4 million units per dose). She demonstrated an excellent clinical response, with complete resolution of the lesions after the third injection (Fig. 3). At the four-month follow-up, the VDRL titer had decreased from 1/32 to 1/8, confirming an adequate therapeutic response.

## Discussion

Primary syphilis usually presents as a painless chancre that often goes unnoticed, leading many patients to seek care only during the secondary stage, characterized by a non-painful, reddish or copper-colored macular rash involving the palms, soles or the trunk [2].

Syphilis is referred to as the "great imitator" because of its wide range of clinical manifestations [3]. Granulomatous inflammation with lymphohistiocytic nodules and plasma cells, as observed in this case, represents an uncommon histopathological feature of secondary syphilis [1, 4].

Unlike the more common forms of secondary syphilis, granulomatous syphilis usually presents as asymptomatic, persistent erythematous papules or nodules, typically sparing the palms and soles. These lesions are most frequently found on the head, neck, trunk, and extremities, further complicating the diagnosis. This atypical presentation therefore represents an additional diagnostic challenge.

Another unusual manifestation of secondary syphilis is the sporotrichoid pattern, which is thought to result from the lymphatic spread of *T. pallidum*. In contrast to other sporotrichoid conditions that typically affect the extremities and may or may not be associated with lymphadenopathy, sporotrichoid secondary syphilis usually involves the postauricular, occipital, and posterior cervical lymph nodes.

The differential diagnosis of secondary syphilis includes conditions such as psoriasis, viral exanthems, cutaneous lymphoma, granuloma annulare, and sarcoidosis [3, 5]. Because of its broad spectrum of clinical and histopathologic manifestations, syphilis is aptly termed the "great mimicker." Histologically, a plasma cell–rich dermal infiltrate is common, while granuloma formation with a lymphohistiocytic infiltrate accompanied by plasma cells is rare. Recognizing these histopathological findings is crucial to establishing an accurate diagnosis of secondary syphilis.

Serologic testing remains the gold standard for screening and diagnosing syphilis. While immunohistochemical detection of *T. pallidum* is available, it can be costly and is not always accessible. Benzathine penicillin remains the treatment of choice at all stages of syphilis.

## Conclusion

This case of nodular secondary syphilis with granulomatous inflammation highlights the broad clinical and histopathological spectrum of syphilis. The unusual presentation of persistent erythematous papules and nodules sparing the palms and soles reinforces its reputation as the "great imitator." Such variability poses significant diagnostic challenges and underscores the importance of maintaining a high index of suspicion supported by serologic confirmation. Benzathine penicillin remains the first-line therapy across all stages of the disease.
